# Ultrasound-guided sclerotherapy with 76% meglumine diatrizoate for bilateral chylothorax after thyroid cancer surgery: a case report

**DOI:** 10.3389/fonc.2025.1684418

**Published:** 2026-01-07

**Authors:** Qinhui Luo, You Peng, Liangling Lao, Xiaolei Hu, Jianlong Xie, Jianming Luo, Hongjie Luo, Liyao Lin

**Affiliations:** Department of Thoracic Surgery, Affiliated Hospital of Guangdong Medical University, Zhanjiang, China

**Keywords:** bilateral chylothorax, case report, meglumine diatrizoate, postoperative complication, thyroid cancer

## Abstract

Bilateral chylothorax following thyroid cancer surgery is exceedingly rare, and management becomes more challenging when one side presents as high-output (>1,000 mL/day). Conservative treatments often yield limited success. We report a case of recurrent papillary thyroid carcinoma in which bilateral chylothorax was diagnosed on postoperative day (POD) 5 by chest radiography and qualitative chyle tests, with right-sided drainage exceeding 1,000 mL/day. After 19 days of ineffective conservative therapy, 76% meglumine diatrizoate was percutaneously injected into the lymphatic leak site under ultrasound guidance, followed by external compression. The drainage volume decreased significantly and fully resolved within 10 days, without systemic adverse effects. To our knowledge, this is the first report of successful treatment of bilateral chylothorax after thyroidectomy using this approach. The findings suggest that local sclerotherapy with meglumine diatrizoate offers a minimally invasive, safe, and technically simple alternative, particularly for patients who fail conservative therapy but are not suitable surgical candidates.

## Introduction

Chylothorax is a rare complication following cervical lymph node dissection for thyroid cancer, with an estimated incidence of approximately 0.29% (1). When the thoracic duct or its branches are injured, chyle may leak through cervical or mediastinal fascial planes into the pleural cavity, resulting in nutritional depletion, immunosuppression, and respiratory compromise. Management becomes particularly challenging in cases of bilateral or high-output chylothorax (2). Traditional conservative therapies, such as fasting and somatostatin infusion, often show limited efficacy. Although surgical thoracic duct ligation may be effective, it carries the risk of disrupting lymphatic return (3). In recent years, thoracic duct embolization (TDE) has emerged as a promising interventional approach, but its technical complexity and reliance on specialized imaging and equipment limit its broad applicability (4). This case report is the first to describe successful closure of the lymphatic leak in a patient with postoperative bilateral chylothorax using ultrasound-guided local injection of 76% meglumine diatrizoate. The procedure was effective and supports the feasibility of this method as a precise, minimally invasive, and technically straightforward treatment option.

## Case report

**Figure 1:**
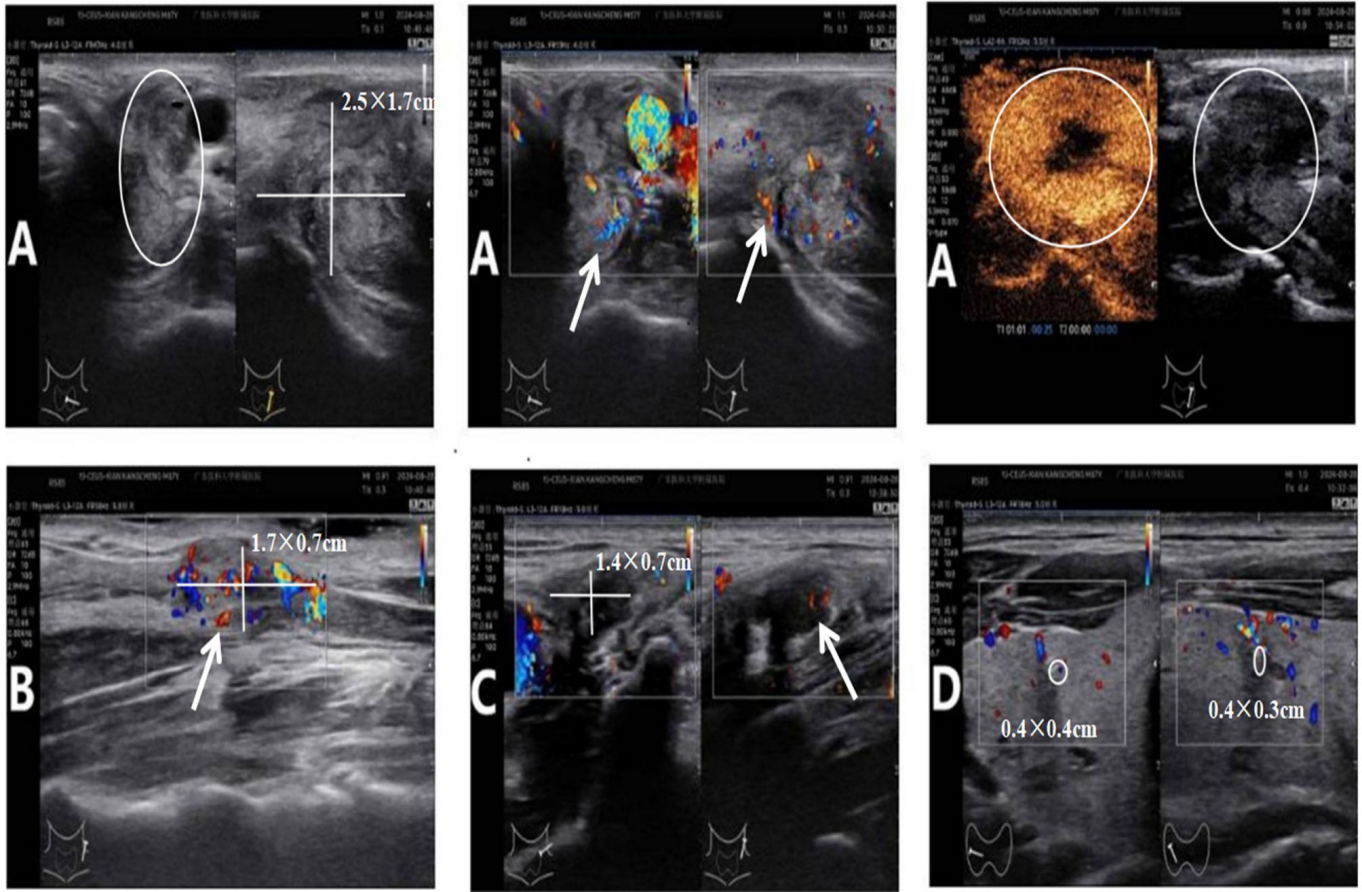
Preoperative contrast-enhanced cervical ultrasound images. Symbol key: white arrows = blood-flow signals on Doppler/CEUS; white circles/ellipses = outlined lesion; white crosshair calipers = linear measurements shown on the panel. **(A)** Two orthogonal views of a hypoechoic lesion in the left thyroid bed; the lesion is outlined by a white ellipse, and white crosshair calipers show 2.5 × 1.7 cm (third dimension 2.2 cm by orthogonal view); color Doppler views of the same lesion, with white arrows indicating blood-flow signals around/within it; contrast-enhanced images with the lesion circled in white. **(B)** A hypoechoic lesion located superolateral to the left common carotid artery; white arrows mark blood-flow signals, and white crosshair calipers show 1.7 × 0.7 cm. **(C)** Multiple hypoechoic lesions in left level II with partial fusion; white crosshair calipers show 1.4 × 0.7 cm for the index cluster, and a white arrow marks representative blood-flow signals. **(D)** Small solid hypoechoic lesion(s) in the right thyroid lobe (white circles), with the displayed sizes 0.4 × 0.3 cm and 0.4 × 0.4 cm.

**Figure 2:**
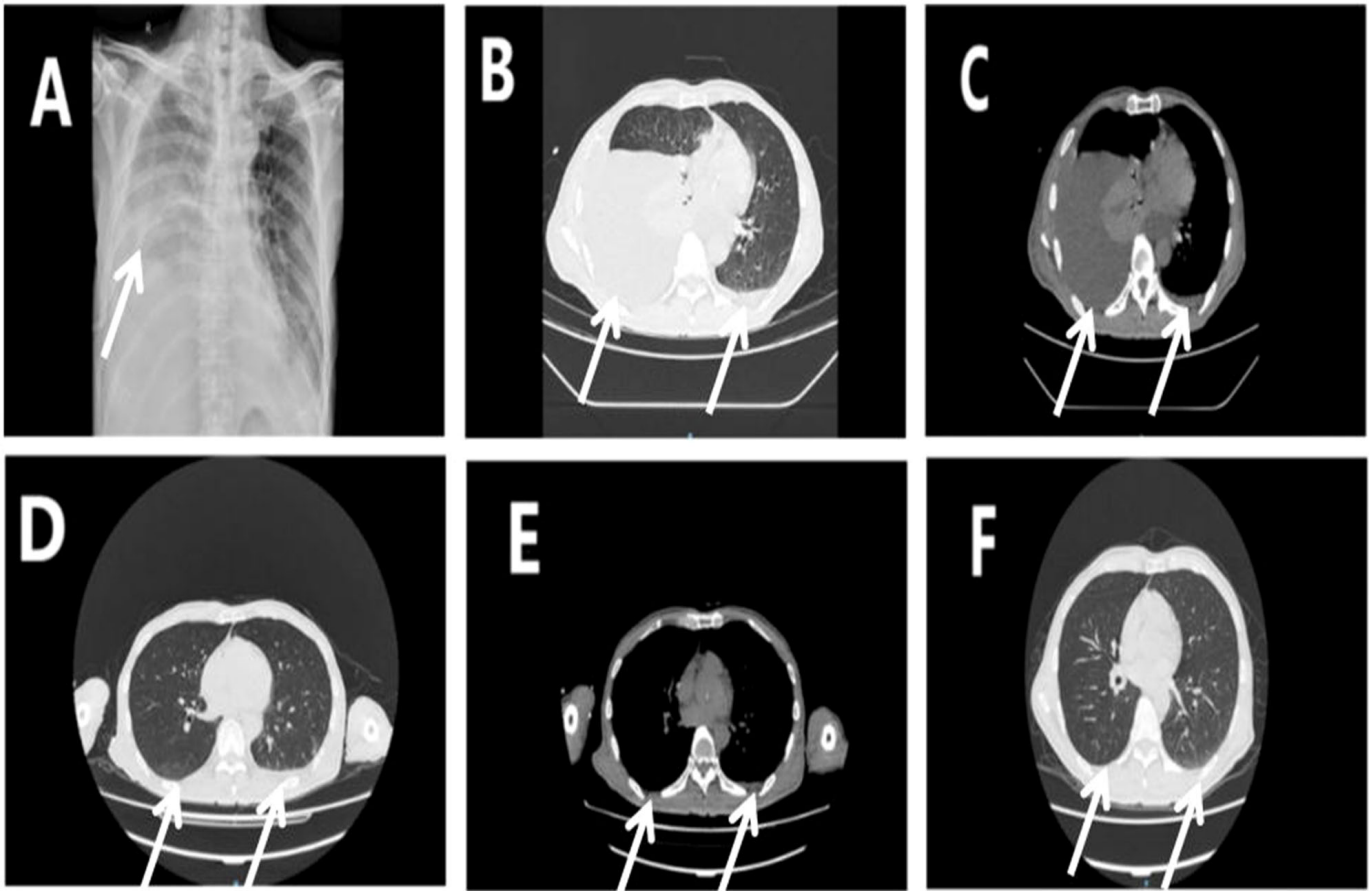
Radiologic changes of the chest before and after treatment. **(A)** Chest X-ray on POD 5 showing massive right pleural effusion (white arrow). **(B, C)** Chest CT on POD 14: lung window **(B)** and mediastinal window **(C)** showing massive right and mild left pleural effusions (white arrows). **(D, E)** Chest CT on POD 35: lung window **(D)** and mediastinal window **(E)** showing significant reduction in right-sided effusion (white arrows). **(F)** Chest CT at 3-month follow-up (lung window) showing near-complete resolution of bilateral pleural effusions (white arrows).

**Figure 3:**
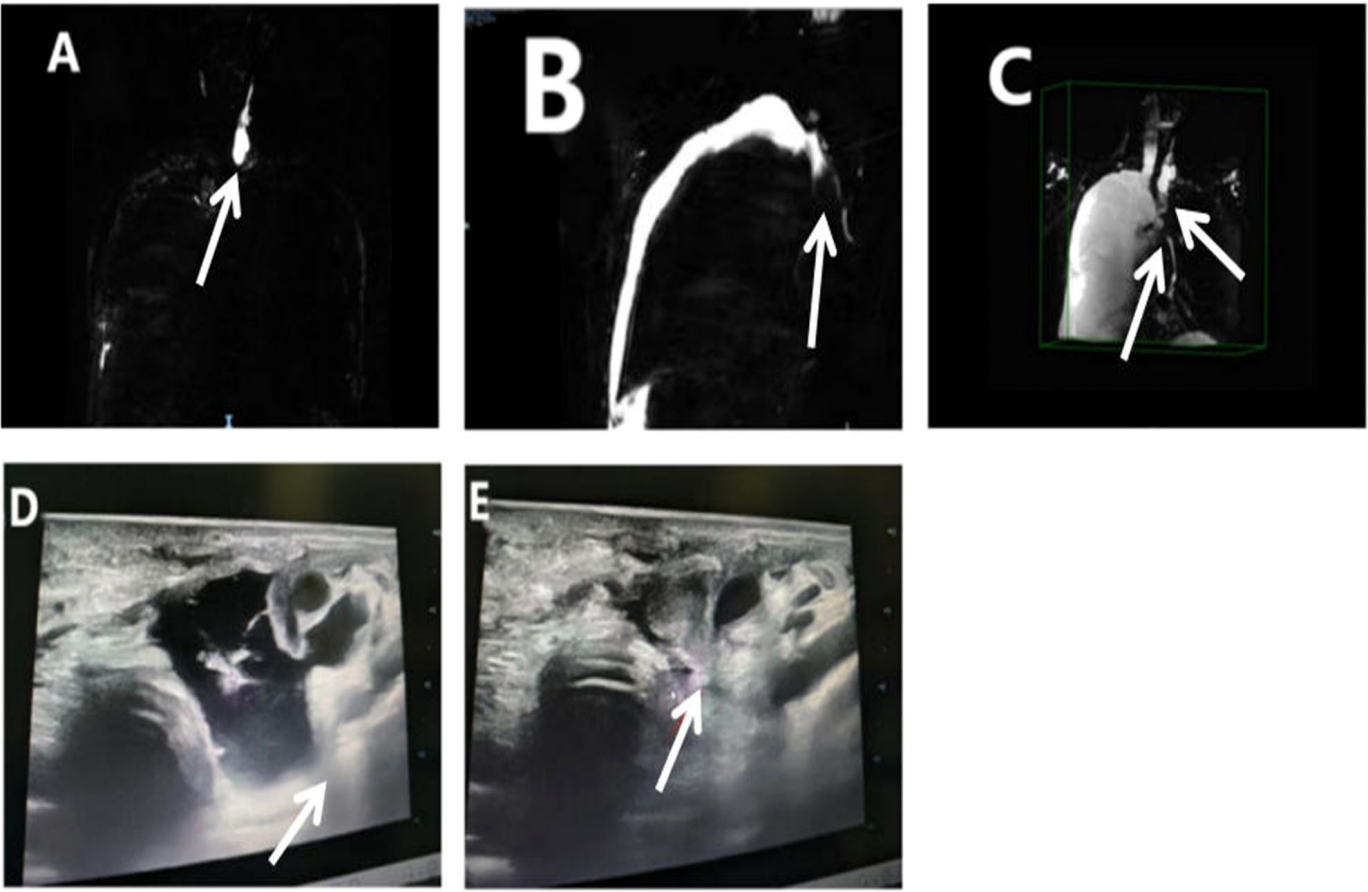
Lymphangiography, MRI, and ultrasound-guided intervention. **(A)** T2-weighted MR lymphangiography showing fluid accumulation in the left thyroid bed with communication to the left cervical lymphatic trunk (white arrow). **(B, C)** Coronal and 3D reconstructed images demonstrating communication between a right thoracic duct branch and the right pleural effusion (white arrows). **(D)** Pre-puncture ultrasound image revealing cervical lymphatic fluid collection and a suspected leak channel (white arrow). **(E)** Under real-time ultrasound guidance, 76% meglumine diatrizoate was locally injected at the leak site (needle tip indicated by the white arrow).

**Figure 4:**
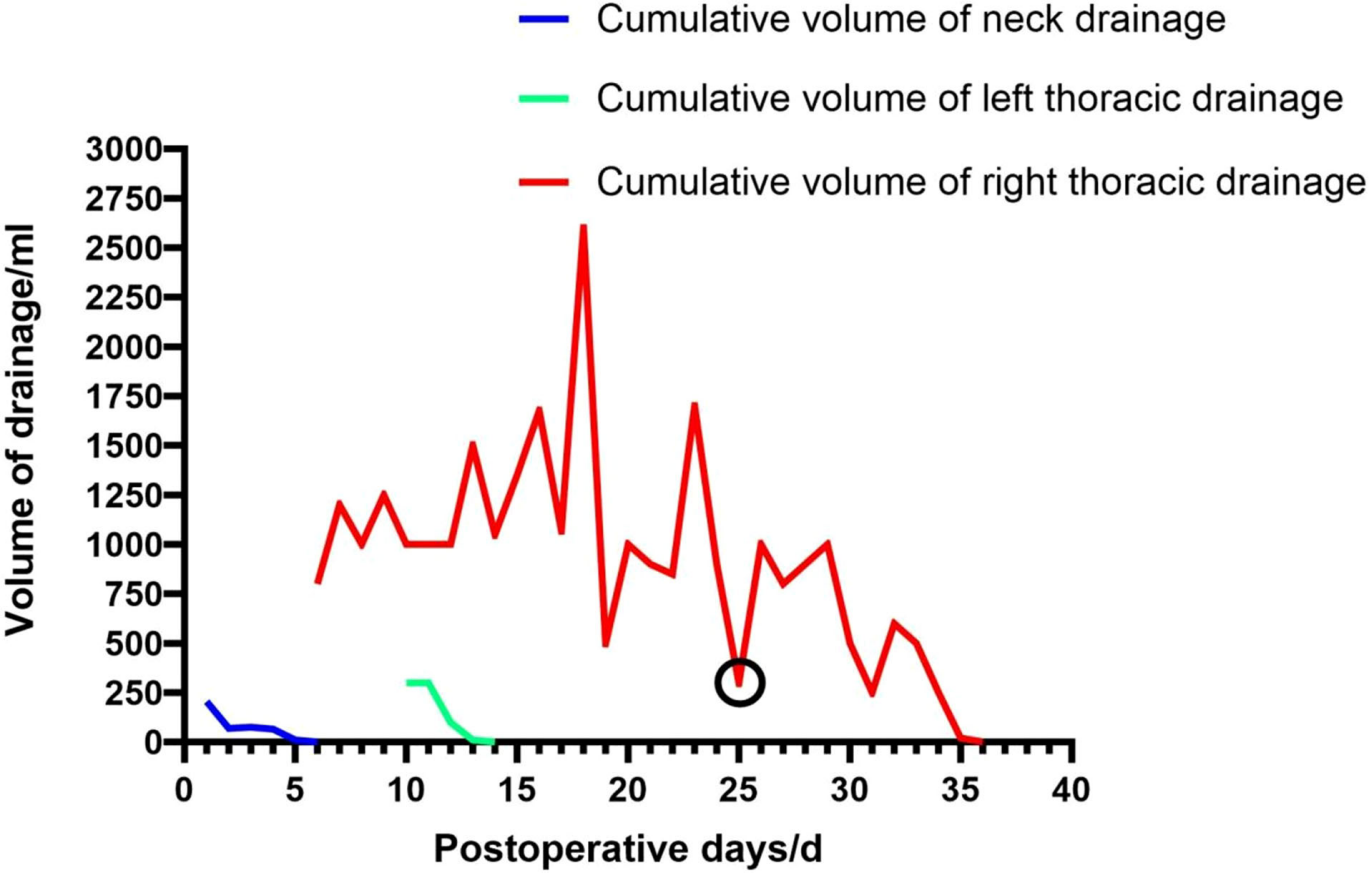
Postoperative cervical and pleural drainage trends. The graph illustrates daily drainage volumes from the cervical region (blue), left pleural cavity (green), and right pleural cavity (red). On POD 18, right-sided drainage peaked at 2,600 mL/day. The black circle indicates the day of ultrasound-guided injection of meglumine diatrizoate, after which the drainage volume declined markedly and nearly resolved by POD 35.

In September 2024, a 67-year-old man was admitted for evaluation of a right thyroid nodule. Ten years earlier, he had undergone left lobectomy and isthmectomy for papillary thyroid carcinoma (PTC). At admission, he was asymptomatic and denied any family history of cancer or exposure to carcinogens. Physical examination revealed a solitary, firm, mobile, and non-tender nodule in the left anterior neck. Contrast-enhanced thyroid ultrasound (**Figure 1**) showed a hypoechoic mass (2.5 × 1.7 × 2.2 cm) in the left thyroid bed with poorly defined margins, and a solid nodule (0.4 × 0.3 × 0.4 cm) in the mid-right thyroid lobe. Fine-needle aspiration (FNA) of the right nodule revealed a benign follicular lesion (Bethesda category II), while the left lesion was confirmed as recurrent PTC. The patient subsequently underwent completion thyroidectomy and bilateral cervical lymph node dissection. Intraoperatively, the left tumor was found to invade the superficial muscle layer of the esophagus and the left recurrent laryngeal nerve, which was resected. The right recurrent laryngeal nerve was preserved. Drains were placed in the thyroid bed and bilateral cervical regions. Postoperative histopathology confirmed recurrent PTC with a positive BRAF V600E mutation. Initial recovery was uneventful. On POD 5, the patient developed sudden chest tightness and dyspnea (SpO_2_ 92% on room air). Chest X-ray (**Figure 2**) revealed massive right pleural effusion. Closed thoracic drainage was performed, yielding 800 mL of milky yellow fluid. Pleural fluid analysis was positive for chyle (Sudan III and Rivalta test), with no evidence of infection. Conservative treatment, including fasting, total parenteral nutrition (TPN), and continuous somatostatin infusion, was initiated immediately. By POD 10, bilateral pleural effusions had worsened. The right drainage volume reached 1,000 mL/day, while the left peaked at approximately 300 mL/day. The left effusion resolved under conservative management, but the right effusion persisted as high-output chylothorax (>1,000 mL/day). By POD 18, the peak negative-pressure drainage reached 2,600 mL/day, adversely affecting nutritional and immune status. A non-suction collection bag was then used to cap daily drainage at approximately 1,000 mL; brief negative-pressure drainage was allowed if chest discomfort occurred. Chyle testing remained positive, consistent with refractory high-output chylothorax. On POD 24, lymphangiography and MRI (**Figure 3**) demonstrated lymphatic leaks from the left cervical region and a branch of the right thoracic duct. On POD 25, under ultrasound guidance, a 10-mL syringe was used to percutaneously access the cervical fluid cavity; 20 mL of chylous fluid was aspirated, recurrent leakage from the tract was observed, and an additional 15 mL was aspirated. Subsequently, 15 mL of 76% meglumine diatrizoate was injected precisely at the leakage point, followed by external compression bandaging. Over the next 10 days, the drainage volume decreased steadily to less than 20 mL/day. The right chest tube was removed on POD 36, and the patient was discharged on POD 38. The postoperative cervical and thoracic drainage trends are shown in (**Figure 4**). At 3-month follow-up, no residual effusion or tumor recurrence was observed on imaging.

## Discussion

Thyroid cancer surgery carries a risk of chyle leakage because cervical lymphatic anatomy is complex and highly variable; injury to the thoracic duct or its cervical tributaries may allow lymph to track along cervical fascial planes into the mediastinum and pleural cavity, resulting in chylothorax (5). In the present case, bilateral chylothorax developed postoperatively with persistent high-output drainage on the right that remained uncontrolled after 19 days of optimized conservative care, indicating the need for a more targeted, minimally invasive intervention. Recent reports suggest that when conservative therapy fails or thoracic duct embolization (TDE) is infeasible or unsuccessful, image-guided, leak-directed sclerotherapy (direct puncture of the leakage point) can effectively control refractory chylothorax or lymphatic leaks (6, 7). Based on this rationale, we innovatively performed ultrasound-guided local sclerotherapy using 76% meglumine diatrizoate. Meglumine diatrizoate is a high-osmolar iodinated contrast agent; local administration creates a hypertonic milieu that induces aseptic inflammation and fibrosis, thereby sealing the fistulous tract—mechanistically supporting its role as a focal sclerosing agent (8). Under real-time ultrasound guidance, we percutaneously accessed the cervical collection, aspirated 35 mL of chyle, and injected 15 mL of 76% meglumine diatrizoate precisely into the suspected leak while avoiding vessels and nerves. This bedside, reproducible technique achieves focal closure with preservation of thoracic duct patency. Drainage declined rapidly after injection, and follow-up imaging demonstrated durable resolution. Concurrently, percutaneous sclerotherapy for postoperative lymphorrhea/lymphocele and lymphatic malformations (e.g., doxycycline, OK-432, ethanol) has an established safety and efficacy profile (9, 10). Case experience also supports the use of meglumine diatrizoate to control lymphatic leaks at other postoperative sites, providing translational justification for this strategy (11).

Our study is innovative in that, for localizable and reachable cervical/upper mediastinal leak tracts, we performed real-time ultrasound–guided focal sclerotherapy using 76% meglumine diatrizoate as a high-osmolar sclerosing agent, achieving precise closure of the leak itself while preserving patency of the main thoracic duct; this approach complements existing pathways in both strategy and technical accessibility. It differs fundamentally from prior treatments: pleurodesis acts within the pleural cavity—via chemical agents (e.g., talc, bleomycin) or mechanical abrasion—to create parietal–visceral pleural adhesion and obliterate the pleural space, thereby controlling intrapleural chyle accumulation, but it targets the pleural cavity rather than the lymphatic leak or the thoracic duct and therefore does not directly repair the primary source of duct/branch leakage; pleurodesis is repeatedly applied in refractory chylothorax or malignant effusions with established mechanisms and efficacy (12). In contrast, thoracic duct embolization (TDE) targets the etiologic level: after percutaneous intranodal lymphangiography, the thoracic duct is catheterized, with occlusion of the duct and/or the leak. Although widely accepted as a minimally invasive technique with high success rates, TDE depends on access route, equipment, and operator expertise and, despite a low overall complication rate, has been associated with access-related events (e.g., intra-abdominal bleeding; perihepatic or para-aortic hematoma with transabdominal or transbiliary/transpancreatic routes; occasional bile peritonitis requiring surgical or interventional management; pancreatitis after pancreatic traversal; and pneumothorax with transpulmonary access), material misdeployment or migration (most commonly unintended migration of adhesive agents, with rare portal-vein embolization), and long-term functional sequelae due to altered lymphatic return (e.g., chronic diarrhea, lower-extremity edema, abdominal distension) (13, 14). We did not pursue surgical ligation of the thoracic duct because imaging localized cervical/upper mediastinal leakage pathways that were safely accessible under ultrasound guidance (on the left, communicating with the left jugular trunk; on the right, a superior thoracic-duct branch communicating with the right pleural cavity). Surgical ligation typically entails transection of the main thoracic duct, carries greater morbidity, may compromise physiological lymphatic return, and may fail to address multiple or branch-level leaks in a single operation.

With regard to safety and indications, this approach uses a small, locally administered dose of a hyperosmolar iodinated contrast agent for sclerotherapy; under strict ultrasound guidance, slow extravascular injection with subsequent local compression and observation can keep the risks of local pain, soft-tissue irritation/inflammation, and subcutaneous extravasation within an acceptable range. It should be avoided or used with caution in patients with a history of iodinated-contrast allergy, severe renal impairment, pregnancy, or an unsafe puncture window. Candidate indications include persistent or recurrent leakage despite adequate conservative therapy; imaging-defined, focal and ultrasound-reachable leaks or communications; and scenarios in which thoracic duct embolization (TDE) or surgery is contraindicated, unavailable, or declined. Limitations include the single-case nature, short- to mid-term follow-up, and off-label use. Prospective multicenter studies are warranted to determine the optimal dose and number of sessions, establish long-term safety, enable head-to-head comparisons with TDE and surgical ligation, and refine patient selection and procedural pathways across diverse etiologies and anatomic patterns of chylothorax.

## Conclusion

To our knowledge, this is the first report of successful use of meglumine diatrizoate for bilateral chylothorax after thyroidectomy. In patients with conservative-therapy failure and a localizable, reachable leak, ultrasound-guided focal sclerotherapy with 76% meglumine diatrizoate offers a minimally invasive, duct-sparing, and resource-friendly option that complements thoracic duct embolization (TDE) and surgery. These findings support further dissemination and prospective validation of this approach.

## Data Availability

The original contributions presented in the study are included in the article/supplementary material. Further inquiries can be directed to the corresponding author.
